# Reviews and Book Notices

**Published:** 1882-06

**Authors:** 


### Reviews and Book Notices.

---

ARTICLE VII.—HOMŒOPATHY.—WHAT IS IT? A STATEMENT AND REVIEW OF ITS DOCTRINES AND PRACTICE. By A. B. PALMER, M.D., LL.D., Professor of Pathology and Practice of Medicine in the College of Medicine and Surgery in the University of Michigan.

This little *brochure* consists of the subject matter of a series of lectures delivered before the students of Ann Arbor University, containing an exposition of the Homœopathic faith and a fair discussion of its doctrines.

The writer asserts that the kind of opposition hitherto made to Homœopathy gives the non-medical public an impression that there exists an unreasonable prejudice against a body of professional men, and a narrow illiberality toward a system of medicine professing to be an improvement on the ordinary methods of the regular practitioners; and this gives the Homœopathic fraternity an opportunity to appeal to public sympathy by the cry of persecution for opinion's sake.

The laity have usually extremely vague ideas on the subject of medicines and the different medical schools. They have an indistinct impression that there is some specific system of old school medicine called Allopathy opposed to and intolerant of the new school system of Homœopathy, but are without any definite or technical knowledge of either, they are ignorant of the fact that regular scientific medicine is no exclusive, dogmatic system at all. They do not understand that it is a science and an art. A system of scientifically observed facts, with certain practical deductions drawn from them without speculative dogma or canon of faith. They usually become adherents of one or the other class of practitioners from some accidental influence, or from crude inexact observations they may have made, but without any fair examination of the subject.

The general object of this little work is to supply one of our defects in the treatment of the Homœopathic system. There is no temptation to exaggerate the peculiarities of these teachings, as they are sufficiently striking in the language of their authors. Pains have been taken to give exact quotations, and to present the unmistakable meaning of the quoted text. The references to titles and pages of Homœopathic works are made with care.

The exact status of Homœopathy in England and on the Continent, ascertained by personal inquiry, is given from the lips of many prominent European medical men. It is stated by them that the chief supporters of the waning system are among the country clergy, who, provided with hand-books and medicines, dose their parishioners for their minor ailments.

The usual absurdities of the so-called provings are related. Tables of the triturations in detail are carried out. Some of them are by Sir James Simpson, of Edinburg, confirmed by the mathematical faculty of Edinburg, which demonstrate that the fifteenth trituration of a grain of medicine would require a mass of sugar sixty-one times as large as the earth. One minim of Mother Tincture, at the twelfth attenuation, demands a sea of water six times the size of the Mediterranean.

To the medical man beset by blatant Homœopathic associates, this little work proves a constant friend. It should be in every practitioner's library. W. L. D.

ARTICLE VIII.—THE MOTHER'S GUIDE, in the Management and Feeding of Infants. By JOHN M. KEATING, M.D., Lecturer on Diseases of Children at the University of Pennsylvania, etc.

This pleasant little volume contains a great deal of common sense advice in plain, clear language, addressed to the better class of mothers. The chapters are mostly on hygienic subjects connected with infantile life from birth to the end of dentition. Those on selection and preparation of artificial foods and the management of constipation, the directions for procedure in the various emergencies of childhood, what to do until the physician arrives, are all excellent. We cordially commend the little book as a companion to all reading mothers. Philadelphia: H. C. Lea's Son & Co. 1881. W. L. D.

ARTICLE IX.—THE ANATOMIST. A Complete Description of the Anatomy of the Human Body. Intended for the Use of Students preparing for Examination. By W. W. HILLES, formerly Lecturer on Anatomy at the Westminster Hospital School of Medicine.

This compact volume is, as it professes to be, a compendium of anatomical information.

The cuts, though small, are the best and clearest we have seen in a volume of this size. Those especially of the ear, eye and joints are fine. The arteries traced in red, the veins in blue, on the limbs outlined in white; the nerves in white on black ground, are suited for vivid impression upon the memory. An excellent book for hasty perusal. New York: G. P. Putnam's Sons. 1881.

W. L. D.

ARTICLE X.—ILLUSTRATIONS OF DISSECTIONS, VOL. II. A Series of Original Colored Plates the Size of Life. By G. V. ELLIS and G. H. Ford.

Reduced on a uniform scale and roproduced in fac simile. The drawings from nature by Mr. Ford, from dissections by Professor Ellis, of University College, London. This forms another of Wood's fine series for 1882.

The accompanying text in some places treats of the operative surgery of the parts illustrated, and furnishes a very acceptable demonstration. Wm. Wood & Co., New York: 1882.

W. L. D.

ARTICLE XI.—A GERMAN-ENGLISH DICTIONARY OF WORDS AND TERMS USED IN MEDICINE AND ITS COGNAGTE SCIENCES. By FANCOURT BARNES, M.D., M.R.C.P., Lord Physician to the Lying-in Hospital, etc. Philo P. Blakiston.

The writer has endeavored to translate most of the words used in botany, chemistry, anatomy, physiology, medicine and surgery.

Such a work, in view of the ever-increasing number of new words, needs constant revision to be of much service to the student. The volume is of handy size, and will form a valuable companion to the translator. W. L. D.

ARTICLE XII.—MANUAL OF THE PHYSICAL DIAGNOSIS OF DISEASES OF THE HEART, Including the Use of the Sphygmograph and Cardiograph. By ARTHUR ERNEST SANSOM, M.D., London.

This is a most valuable little volume. The author has exhausted the subject of the physical diagnosis of diseases of the heart. Some of his statements are too dogmatical; as "notable feebleness of impulse, co-existing with irregularity, is pathognomonic of dilatation of the left ventricle, and indicates that the muscular fibers have become disintegrated, and have lost their tonicity."

The author has investigated the use of the sphygmograph and cardiograph, and has recorded many useful facts. But until these instruments can be simplified and made more uniform in their action, they must be more useful to the clinical investigator than to the every-day practitioner.

ARTICLE XIII.—TRANSACTIONS OF THE INDIANA STATE MEDICAL SOCIETY, AND CODE OF ETHICS, 1881.

This contains the President's address, by Dr. T. B. Harvey, Indianapolis; various papers; transactions of the thirty-first session, constitution and by-laws; with American Code of Ethics.

H. Charles, M.D., of Carthage, in a paper on "Tobacco and its Toxic Effects," says: "I am led to the conclusion that tobacco is the cause of more heart disease than any one thing beside; and I am inclined to think that it brings on more of this trouble, in its various phases, than all other causes combined." This is diametrically opposed to the teachings of Flint, Fothergill and others.

ARTICLE XIV.—REPORT OF STATE BOARD OF HEALTH, MICHIGAN, 1880.

From this report it is evident that the most widely distributed, although not often fatal, disease is intermittent fever; 92 per cent. of the diseases observed and reported being intermittent fever. Of other diseases, 90 per cent reported diarrhœa; 32 per cent. diphtheria; 10 per cent. scarlet fever. No small-pox was reported from any part of the State. Contributions by John Mulvey, M.D., British Navy, and A. W. Nicholson, M.D., Otisville, on "Ozone in Nature," and the best methods of observing it, are quite instructive.

TRANSACTIONS MISSOURI STATE MEDICAL ASSOCIATION, 1880.

SECOND ANNUAL REPORT OF BOARD OF HEALTH, LUNACY AND CHARITY, SUPPLEMENTARY, OF MASSACHUSETTS, 1880.

